# Effects of strain relaxation in Pr_0.67_Sr_0.33_MnO_3_ films probed by polarization dependent X-ray absorption near edge structure

**DOI:** 10.1038/srep19886

**Published:** 2016-01-28

**Authors:** Bangmin Zhang, Jingsheng Chen, Ping Yang, Xiao Chi, Weinan Lin, T. Venkatesan, Cheng-Jun Sun, Steve M. Heald, Gan Moog Chow

**Affiliations:** 1 Department of Materials Science & Engineering, National University of Singapore, 117576, Singapore; 2 Singapore Synchrotron Light Source (SSLS), National University of Singapore, 5 Research Link, 117603 Singapore; 3 Department of Physics, National University of Singapore, 117542, Singapore; 4 NUSNNI-Nanocore, National University of Singapore, 117411, Singapore; 5 Department of Electrical & Computer Engineering, National University of Singapore, 117576, Singapore; 6 Advanced Photon Source, Argonne National Laboratory, Argonne, IL 60439, USA

## Abstract

The Mn ***K*** edge X-ray absorption near edge structure (XANES) of Pr_0.67_Sr_0.33_MnO_3_ films with different thicknesses on (001) LaAlO_3_ substrate was measured, and the effects of strain relaxation on film properties were investigated. The films showed in-plane compressive and out-of-plane tensile strains. Strain relaxation occurred with increasing film thickness, affecting both lattice constant and MnO_6_ octahedral rotation. In polarization dependent XANES measurements using in-plane (parallel) and out-of-plane (perpendicular) geometries, the different values of absorption resonance energy ***E***_r_ confirmed the film anisotropy. The values of ***E***_r_ along these two directions shifted towards each other with increasing film thickness. Correlating with X-ray diffraction (XRD) results it is suggested that the strain relaxation decreased the local anisotropy and corresponding probability of electronic charge transfer between Mn 3*d* and O 2*p* orbitals along the in-plane and out-of-plane directions. The XANES results were used to explain the film-thickness dependent magnetic and transport properties.

The perovskite manganites (Re_1-x_*A*_x_MnO_3_), where *Re* is a trivalent rare earth, and *A* is a divalent metal exhibit a range of interesting properties^1^,^2^ such as high spin polarization and colossal magnetoresistance (CMR). Manganites are potential candidates for resistance random access memories (RRAM) in the next generation of non-volatile memories^3^,^4^,^5^. The electronic structure plays an important role^4^ in resistance switching of RRAM^6^,^7^,^8^. The electronic structure of manganite is correlated with the local environment^9^ that affects material properties^10^. For example, the intersite hopping of Mn 3*d* electrons through the bridging oxygen 2*p* orbital affects the double-exchange coupling strength^11^ and Curie temperature ***T***_c_. Crystal structure and electronic structure are correlated through strong electron-phonon coupling in manganites^1^. Thin films grown on single crystal substrates are normally strained due to lattice-mismatch between the film and substrate. The crystal structure and octahedral rotation of thin films may therefore differ from that of bulk^12^. Strain relaxation occurs gradually and continuously with increasing film thickness, leading to film-thickness dependent ***T***_c_^1^,^9^,^12^. Polarization dependent X-ray absorption structure (XAS) may be used to probe the film-thickness dependent electronic structure. Correlating the electronic structure with the crystal structure, material properties such as magnetoresistance may be better understood.

X-ray absorption near edge structure (XANES) provides insight into the electronic structure of the absorbing atom^13^. For manganites, the absorbing ion of interest is Mn in the MnO_6_ octahedron. The Mn XAS may be collected either by total electron yield (TEY) mode or total fluorescence yield (TFY) mode^14^. Due to the low fluorescence signal intensity in the soft X-ray region^15^, the TFY mode cannot be used. Instead the total electron yield (TEY) mode may be employed to collect the Mn ***L*** edge (~640 eV) XAS, however, the signal sampling depth is limited to only a few nanometers near the surface^10^,^14^. The surface-sensitive information from TEY may not necessarily be representative for thick films. Additional information and discussion of Mn ***L*** edge data are found in Supplementary Figure S1 online.

In the hard X-ray region, the quality of the TFY signal is enhanced, and therefore may be used to collect the Mn ***K*** edge (~6.5 keV) signal^16^. The TFY signal is bulk sensitive^17^ and is suitable for investigating the effects of strain relaxation in films of the various thicknesses (12 to 100-nm) as used in our current study. The main absorption structure in the Mn ***K*** edge XANES is due to the excitation of a Mn core 1*s* electron to empty 4*p* orbitals^18^, which is sensitive to the local environment around the absorbing atom. For example, due to hybridization between Mn 3*d* orbitals and O 2*p* orbitals, there exists a mixture of O 2*p* orbitals with Mn 3*d* orbitals. The electronic structure with *n* electrons in Mn 3*d* orbitals may be described as a mixture of two configurations^19^,^20^,^21^,^22^: 3*d*^n^ ***L*** and 3*d*^n+1^
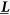
***. L*** refers to the O ligand surrounding the Mn ion, and 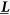 indicates O ligand with one electron hole resulting from the electron transfer from O 2*p* to empty Mn 3*d* orbitals. After X-ray absorption, two electronic configurations exist for the final states: 3*d*^n^***L***4*p*^1^ and 3*d*^n+1^
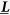
4*p*^1^. These final states may be revealed in the fine structure of XANES. The effects of strain relaxation on film properties may be investigated using the sensitivity of XANES to the crystal structure.

In this work we report the Mn ***K*** edge X-ray absorption near edge structure (XANES) of Pr_0.67_Sr_0.33_MnO_3_ (PSMO) films of different thicknesses on (001) LaAlO_3_ (LAO) substrates. The narrow bandwidth manganite has high strain sensitivity^23^. Bulk PSMO has a smaller bandwidth than that of the La_1-x_Sr_x_MnO_3_ system^1^. Thus, bulk PSMO may find potential application due to its high transition temperature, i.e. its ferromagnetic metal (FM)-to-paramagnetic insulator (PI) phase transition at ~ 300 K^24^,^25^. Our results show that the PSMO films deposited on LAO substrate experienced in-plane compressive strain and out-of-plane tensile strain. In polarization dependent measurements, the in-plane (parallel) and out-of-plane (perpendicular) XANES showed anisotropic properties with different absorption energies ***E***_r_ (peak point). With increasing film thickness, the difference of ***E***_r_ between these two polarization dependent measurements decreased. Correlating with the XRD results, it is suggested that the strain relaxation weakened the local anisotropy and the corresponding probability of charge transfer between Mn 3*d* and O 2*p* along in-plane and out-of-plane directions. This is responsible for the change of ***E***_r_ with increasing film thickness. The transport and magnetic properties also changed with strain relaxation, mediated by the change of electronic structure.

## Results

**Figure 1:**
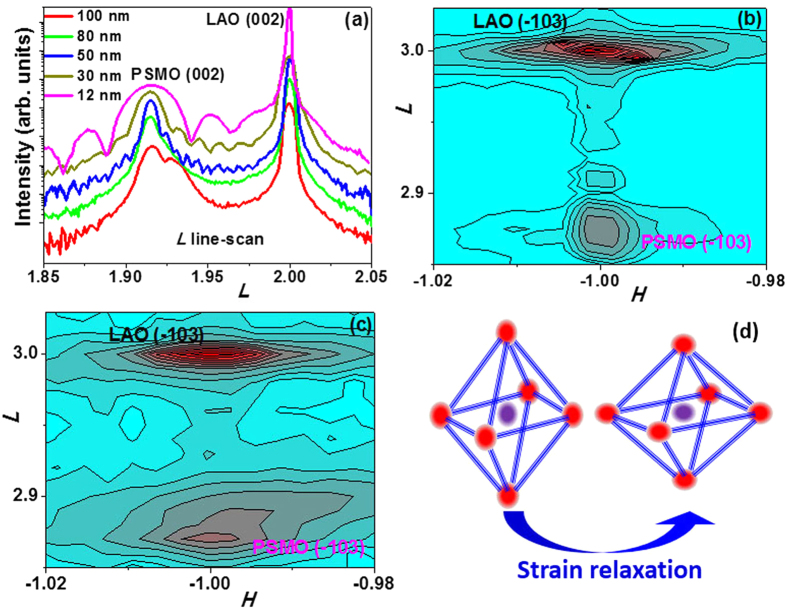
(**a**) (00l) scan of PSMO films with different thicknesses on LAO substrate; reciprocal spacing mapping around (−103) for (**b**) 12-nm and (**c**) 100-nm film; (**d**) illustration of strain relaxation in tetragonal ratio of MnO_6_ octahedron.

The XRD results of PSMO films are shown in Fig. 1(a–c). For the 12-nm film, the in-plane lattice constant (3.790 Å) was the same as that of the LAO substrate (as shown in Fig. 1(b)), indicating that the PSMO film was fully strained by the LAO substrate. The calculated^26^ out-of-plane lattice constant (3.958 Å) from the (002) peak was larger than the bulk value (3.860 Å). The in-plane compressive strain (−1.8%) between the PSMO film and LAO substrate elongated the out-of-plane lattice^27^. In the 80-nm and the 100-nm films, the (00*l*) curves (Fig. 1a) indicate more than one peak. The results were fitted into two components (*P*1 and *P*2) as shown in Supplementary Figure S2 online. The reciprocal space mapping around the (−103) peak and *L* scan indicate that the in-plane and out-of-plane lattice constants are not uniform for the 100-nm film, as a result of strain relaxation in this thicker film (Fig. 1b,c). However, due to the volume-averaged XRD signal and the overlapping peaks, the differentiation of the value of ***H*** for these two parts are difficult. To resolve this challenge, volume conservation was assumed. The in-plane lattice constants of *P*1 and *P*2 components were calculated based on the volume-conservation assumption^28^, as shown in Table 1. Previous work revealed that moving from the LAO substrate into the PSMO film, the ***c*** value of the PSMO film changed gradually from 3.790 Å of substrate to that of PSMO (>3.950 Å) in ~15 unit cells^27^. This could cause a peak shift and broadening. As indicated in Table 1, the lower ***c***value in the 12-nm film compared to that in 30 nm film could be attributed to this non-uniform region. The effects of this region on film properties will be discussed later. With the increase of film thickness from 30 nm, the strain relaxed partially and the out-of-plane lattice parameter ***c*** decreased gradually. Due to strain relaxation, the tetragonal ratio *c*/*a* (Table 1) of the PSMO film changed, and the corresponding change of the MnO_6_ octahedron is illustrated in Fig. 1(d). Previous work of epitaxial films on different substrates^29^,^30^,^31^,^32^ showed that the tetragonal *c/a* ratio had a direct influence on electronic structure and anisotropy in the electronic hopping integral.

**Figure 2:**
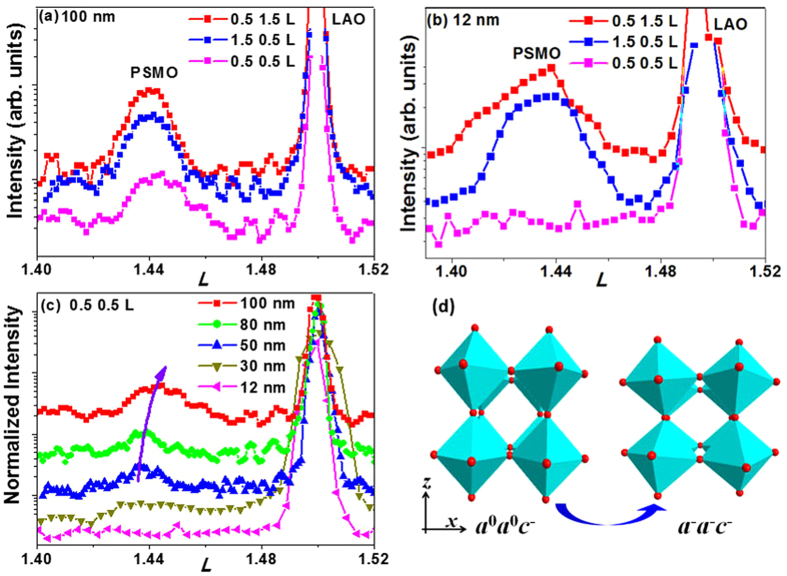
Half-integer diffraction peak of (a) 100-nm and (b) 12-nm PSMO films; (c) the (0.5 0.5 *l*) peak of PSMO film with different thicknesses. The curve was normalized to the LAO (002) peak; (d) illustration of the effect of strain relaxation on MnO_6_ octahedral rotation pattern.

The strain affects not only the lattice constant, but also the MnO_6_ rotation in the PSMO films. In Fig. 2(a–c), half-integer diffraction was measured to determine the octahedral rotation. According to Glazer’s notation^33^,^34^,^35^,^36^, the MnO_6_ rotation pattern in the 12-nm film is ***a***^0^***a***^0^***c***^-^. With increasing film thickness, one additional peak (0.5 0.5 1.5) gradually appeared and became obvious in the 50-nm film, as shown in Fig. 2(c). Note that the intensity of this peak (indicated by the purple arrow in Fig. 2(c) for films ≥ 50 nm) is weak. This suggests that the MnO_6_ rotation in the thick films (≥50 nm) became ***a***^−^***a***^−^***c***^−^. The effects of strain relaxation on the MnO_6_ rotation is illustrated in Fig. 2(d). The measured rotation of AlO_6_ in the LAO substrate was ***a***^-^***a***^-^***a***^-^ in our study, consistent with the reported results^21^. The in-plane compressive strain suppressed the octahedron tilt around the two in-plane (*x* and *y*) axes. The rotation around the two in-plane axes gradually occurred with partial relaxation of in-plane compressive strain in the thick films. This phenomenon is consistent with previous work^21^ that demonstrated the existence of in-plane rotation (***a***^−^/***b***^−^) in strain-free and in-plane tensile strain cases. Based on the integer and half-integer diffraction results, the crystal structure of PSMO films with different thicknesses changed drastically, affecting the corresponding electronic structure and other film properties.

**Figure 3:**
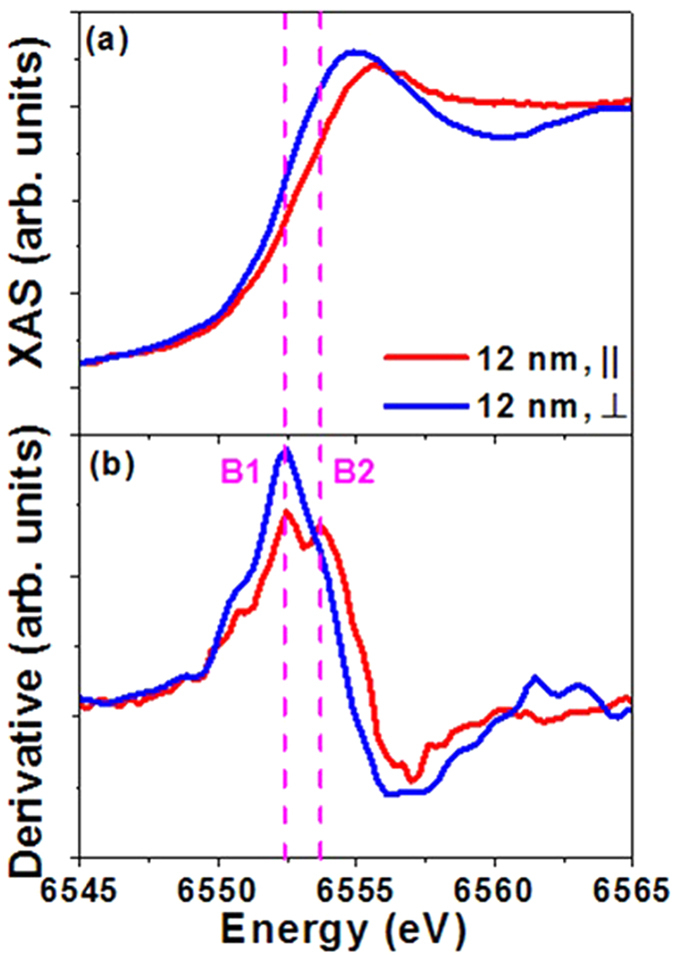
(**a**) Polarized Mn K edge XANES and (**b**) corresponding derivative curves for the 12-nm PSMO film.

The properties of manganites are strongly coupled with the electronic structure. XANES is able to detect the local environment of the absorbing atom and electronic structure. Polarization dependent XANES was used to study the effects of strain on the electronic structure. The polarization dependent XANES is defined as follows: in parallel measurement (||), the polarization vector (***E*** vector) of the X-rays was in the film plane; in perpendicular case (⊥), the polarization vector was perpendicular to the film plane. The polarization-dependent Mn ***K*** edge XANES of the 12-nm PSMO film is shown in Fig. 3. In this study, we only focused on the main absorption edge at ~ 6555 eV, which is due to the electronic excitation from the Mn core 1*s* orbital to empty 4*p* orbitals^18^. The absorption intensity revealed the unoccupied states of the Mn 4*p* orbitals.

**Figure 4:**
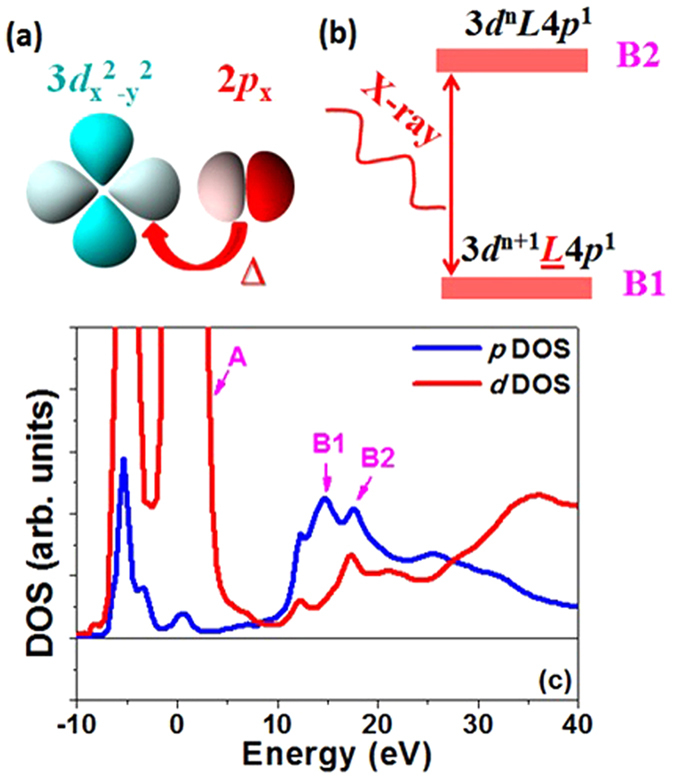
Illustration of (a) charge transfer (∆) between Mn 3*d* and O 2*p* orbitals, and (b) final electronic configuration after X-ray absorption. Refer to the text for details; (c) the calculated *p* and *d*-projected density of states (DOS) of the Mn atom for the 12-nm PSMO film.

As shown in Fig. 3, the resonance energy ***E***_**r**_ (peak point) was different between the two polarization dependent measurements (|| and ⊥). The PSMO film experienced in-plane compressive and out-of-plane tensile strains as shown from the XRD measurements. In the parallel measurement, the polarization direction was along the in-plane compressive strain direction giving a higher ***E***_**r**_ value (6555.8 eV). In the perpendicular measurement, the polarization direction was along the out-of-plane tensile strain direction with a lower ***E***_**r**_ (6554.9 eV). The derivative curves of Mn ***K*** edge XANES are shown in Fig. 3(b). In the parallel measurement, the Mn ***K*** edge XANES revealed two sub-peaks at 6552.4 eV (B1) and 6553.7 eV (B2), respectively. In the perpendicular case, the intensity of the lower energy peak (B1) increased, whereas the higher energy peak (B2) disappeared. There are several possible reasons for the origin of these two sub-peaks. (1) The coexistence of the Mn^3+^ and Mn^4+^ ions in the film^37^,^38^. The ratio of B1/B2 should be similar in both parallel and perpendicular measurements if the ions with different chemical valences are the main factor, whereas our experimental results show different B1/B2 ratios for films with different thicknesses. (2) The energy splitting of Mn empty 4*p*_x_, 4*p*_y_, 4*p*_*z*_ levels due to the anisotropy of the local environment^39^. The strain relaxation in thick film changed its crystal structure, and the position of B1 and B2 should change accordingly if the strain-induced splitting of Mn 4*p* energy level was responsible. However, the positions of B1 and B2 peaks from the derivative curves did not change with different thicknesses. (3) The electronic charge transfer between the Mn 3*d* and O 2*p* orbitals^40^ (Fig. 4(a)). The anisotropic strain along in-plane and out-of-plane directions induces large anisotropy in the local environment in the two directions. The hybridization between Mn 3*d* orbitals and O 2*p* orbitals then becomes anisotropic due to the difference of orbital overlap along these two different directions^41^. During the X-ray absorption process (from Mn core 1*s* to Mn 4*p*), the charge transfer (from O 2*p* to Mn 3*d*) existed simultaneously. The final state may be viewed as a mixture of two electronic configurations^19^,^20^,^21^: 3*d*^n^***L***4*p*^1^, and 3*d*^n+1^
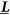
4*p*^1^. The ***L*** refers to the O ligands, and 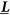 indicates one hole in the O ligands, resulting from the electron transfer from O 2*p* to empty Mn 3*d* orbitals. In the 3*d*^n+1^
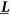
4*p*^1^ configuration, the Coulomb interaction between the Mn 1*s* core hole and the extra 3*d* electron lowers the total energy from electron transfer^15^. Hence, the lower energy B1 peak corresponded to the photon absorption leading to the final configuration 3*d*^n+1^
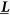
4*p*^1^ as shown in Fig. 4(b), and the higher energy B2 peak corresponds to the photon absorption leading to the final configuration 3*d*^n^***L***4*p*^1^. This assignment was further supported by the density of states (DOS) calculated using the FEFF8.4 code. The 118 atomic cluster was built based on XRD results and the simulation details were expressed elsewhere^42^,^43^. Fig. 4(c) shows the *p, d*-projected density of states (DOS) on the Mn site for a 12-nm PSMO film with the Fermi level set to zero. The peak A in Mn 3*d* orbitals is assigned to the pre-edge of Mn ***K*** edge XANES, while the peak B1 and B2 in *p* orbitals is assigned to the main ***K*** edge absorption^19^. The splitting of the DOS around B1 and B2 causes the multiple peaks in the X-ray absorption curve.

Taking the perpendicular measurement as an example, the excited Mn 1*s* core electron will fill the empty 4*p*_z_ orbital. In this situation, the dominant charge transfer is the in-plane process (Fig. 4(a)), filling the Mn 3*d*_x_^2^_−y_^2^ orbital through O 2*p*_x_, 2*p*_y_ orbitals. Although the Mn 3*d* orbitals hybridized with 6 oxygen atoms in all *x, y, z* directions, the possibility of electron transfer from oxygen 2*p* to fill the Mn 3*d*_3z_^2^_−r_^2^ is limited due to the strong Coulomb interaction between the excited 1s core electron at 4*p*_z_ and the electron at the 3*d*_3z_^2^_−r_^2^ orbital (in the event that the transferred electron occupied the 3*d*_3z_^2^_−r_^2^ orbital). In this case, the in-plane compressive strain enhances the hybridization between Mn 3*d* and O 2*p* orbitals, resulting in strong absorption corresponding to the 3*d*^n+1^
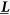
4*p*^1^ (B1 peak) configuration. Similarly, in the parallel measurement, the dominant charge transfer was the out-of-plane process, filling the Mn 3*d*_3z_^2^_−r_^2^ orbital through O 2*p*_z_ orbitals. The out-of-plane tensile strain weakens the hybridization between Mn and O, limiting the absorption corresponding to the 3*d*^n+1^*L*4*p*^1^ (B1 peak) configuration.

**Figure 5:**
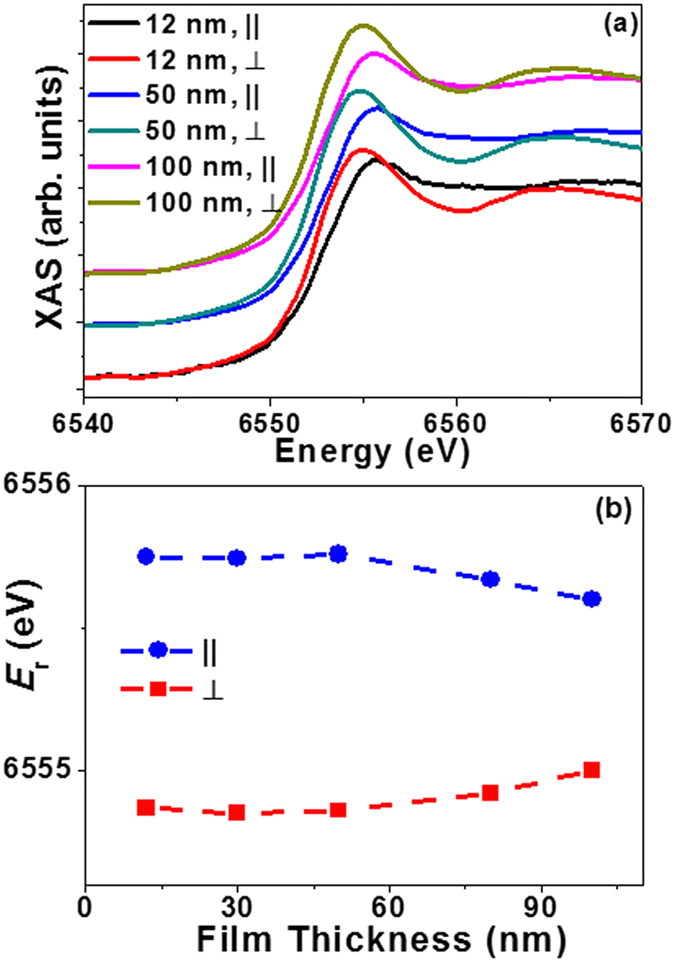
(**a**) Polarized Mn K edge XANES for PSMO films with different thicknesses; (**b**) summary of ***E***_r_ from the parallel and perpendicular measurements with varying film thickness. The dashed lines are viewing guides.

The two B1 and B2 sub-peaks in the derivative curve (Fig. 3d) indicate that the shape of the Mn ***K*** edge XANES has two contributions, and the relative ratio of the B1/ B2 peak intensity affects the maximum position of XANES (***E***_**r**_). Both the crystal structure and the charge transfer were affected by the strain relaxation, which is revealed by ***E***_**r**_. The Mn ***K*** edge XANES of PSMO films of different thicknesses is shown in Fig. 5(a). The difference of ***E***_**r**_ of two polarization dependent measurements, summarized in Fig. 5(b), showed a decrease with increasing film thickness. This phenomenon may be understood as follows: with the existence of strain relaxation, the anisotropy of the local environment along the in-plane and out-of-plane directions decreased as demonstrated in Fig. 1(d). Thus there is a decrease in the difference of probability of electronic charge transfer along the two directions (parallel direction from O 2*p*_x,y_ to Mn 3*d*_x_^2^_−y_^2^; and perpendicular direction from O 2*p*_z_ to Mn 3*d*_3z_^2^_−r_^2^). The difference of relative ratio of B1/B2 peak in XANES is thus decreased, and the resonance energy ***E***_**r**_ along the two directions shifts towards each other with increasing film thickness.

## Discussion

**Figure 6:**
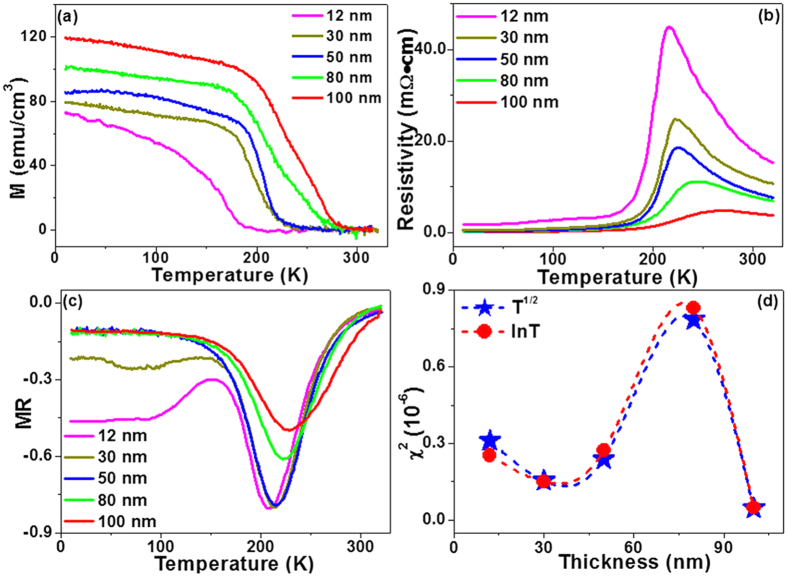
(**a**) the magnetization-temperature (MT) curves of PSMO films with different thicknesses, measured with a field of 100 Oe; (**b**) resistance-temperature (RT) curves at 0T and (**c**) Magnetoresistance (MR) of PSMO film with a magnetic field of 4T; (**d**) The change of χ^2^ with film thickness. The red dot refers to equation (1) and the blue star refers to equation (2). Refer to the text for more information.

The polarization dependent XANES is sensitive to the electronic structure due to the hybridization between Mn 3*d* and O 2*p* orbitals. In the 12-nm film, the in-plane charge transfer predominated over that in the out-of-plane direction. With increasing film thickness, both the in-plane and out-of-plane charge transfer became important. Figure 6(a–c) shows the magnetoresistance (MR = (ρ_H_ − ρ_0_)/ρ_0_) curves for the PSMO films. Large MR values existed near ***T***c in all films. At low temperature the MR value increased with decreasing film thickness. It has been identified that with decreasing film thickness the quantum interference effects (QIE) from electron-electron interactions and weak localization^44^,^45^ become more important at low temperature. The dimensionality of the system, i.e. 3-dimensional (3D) and 2-dimensional (2D), influences the temperature dependent resistance. In the 2D system, the QIE has a logarithmic dependence (ln *T*) as follows^44^,^45^:


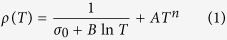


In the 3D system, the QIE changes as *T*^p/2^. The fitting parameter *p* = 1 has been used to determine the change from 3D to 2D^43^, as shown below:


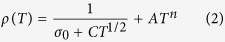


where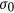, *A, B, C*, and *n* are free fitting parameters. In our work, the low temperature (<60 K) transport data were fitted according to the two above equations. The normalized 
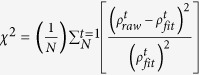
 characterizes how well the experimental data is fitted; a smaller value indicates a better fit. In this equation, *N* is the number of data points, 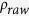 and 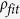 are measured and fitted resistivities, respectively. The fitting details are found in Supplementary Figure S3 online. It was observed that the ln *T* fitting was better with a smaller 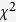 for the 12-nm film; for thicker films (80 nm), the *T*^1/2^ showed a better fit. For the 100-nm film, the absolute difference between the two 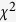 values was very small, probably indicating the decreasing role played by the QIE correction terms in equations (1) and (2)^44^. In other words, the two equations using the QIE correction terms may no longer be applicable in thick films. The fitting data suggest that in the 12-nm film, the 2D QIE seemed to emerge. Figure 6(c) shows an increase of MR value with decreasing film thickness at low temperature (10–100 K). Weak electronic localization in thin film may produce large negative MR effects^46^ at low temperature. Details of the 3D-to-2D cross over as a function of film thickness warrant further study, which is beyond the current scope of investigation.

The double-exchange model suggests that the exchange interaction intensity is proportional to the averaged electron hopping possibility^47^,^48^,^49^,^50^,^51^ between the two neighboring Mn sites (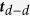) and ***T***_c_. In the case of weak strain, strain relaxation affects the crystal structure and ***T***_c_ of manganite films may be calculated according to the following equation^48^:


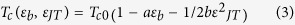


where 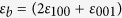 is the bulk strain, 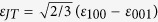 is the Jahn-Teller strain, 
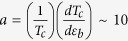
 and 
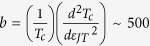
25.

Calculated values of ***T***_c_ using equation (3) increased with increasing film thickness, consistent with the experimental results as shown in Fig. 6(a) and in Table 1. Note that for calculations involving the 80-nm and 100-nm films, the averaged lattice constant was used (from the area ratio of volume-averaged XRD results, see Supplementary Figure S2 online). The discrepancy between experimental and calculated values may arise from ignoring the effects of inhomogeneous crystal structure through film thickness and orbital reconstruction^29^,^30^,^31^,^32^. In the 100-nm film, strain relaxation weakened the anisotropy in the local environment and enhanced the electronic hopping probability. For the 12-nm film with the lowest ***T***_c_, the effects of interface may be important and warrant further investigation.

In summary, the effects of thickness-dependent strain relaxation on Pr_0.67_Sr_0.33_MnO_3_ film properties were investigated. The films showed in-plane compressive strain and out-of-plane tensile strain. Strain relaxation occurred with increasing film thickness, affecting both the lattice constant and MnO_6_ octahedral rotation. These in turn influenced the corresponding electronic structures. In polarization dependent measurements, the in-plane and out-of-plane XANES were anisotropic with different absorption energy ***E***_r_. With increasing film thickness, the difference of ***E***_r_ between the two polarization dependent measurements decreased. Based on the XRD results, the strain relaxation weakened the local anisotropy and probability of charge transfer (between Mn 3*d* and O 2*p* orbitals) along the in-plane and out-of-plane directions, giving rise to the change in ***E***_r_. The magnetoresistance effect and Curie temperature of PSMO films also showed variation with strain relaxation.

## Methods

### Film fabrication

Pr_0.67_Sr_0.33_MnO_3_ (PSMO) films with different thicknesses (12, 30, 50, 80 and 100 nm) were grown on (001) LaAlO_3_ (LAO) single crystal substrates using a 248 nm KrF pulsed laser at a substrate temperature of 780 °C and a pure oxygen pressure of 26 Pa. The energy of the laser beam was 90 mJ and the pulse frequency was 5 Hz. After deposition, the films were cooled to room temperature at 20 K/min.

### Characterization

X-ray diffraction (XRD) was measured using four-circle diffractometer at the Singapore Synchrotron Light Source (SSLS) with Cu*K*_α1_ radiation equivalent. The magnetic properties were measured by a superconducting quantum interference device (SQUID), and the transport properties were measured by Physical Property Measurement System (PPMS). The polarization dependent XANES measurements were performed using linear polarized X-rays at beamline 20-ID-B of the Advanced Photon Source (APS), Argonne National Laboratory, USA. Fluorescent X-rays were detected using a multi-element germanium detector, and the XANES normalization was done using Athena. Details on the beamline optics and instruments may be found elsewhere^25^,^26^. A Mn metal foil was placed to intercept a scattered beam for monochromator energy calibration.

## Additional Information

**How to cite this article**: Zhang, B. *et al*. Effects of strain relaxation in Pr_0.67_Sr_0.33_MnO_3_ films probed by polarization dependent X-ray absorption near edge structure. *Sci. Rep.* **6**, 19886; doi: 10.1038/srep19886 (2016).

## Supplementary Material

Supplementary Information

## Figures and Tables

**Table 1:** Summary of averaged lattice constant, tetragonal ratio, measured (Exp.) and calculated (Cal.) Curie temperature ***T***_c_ of PSMO films with different thicknesses.

**Thickness (nm)**	**a (Å)**	**c (Å)**	**c/a**	**MnO**_**6**_ **Tilt**	**T**_**c**_**/T**_**c, 30** nm_	
					**Exp.**	**Cal.**
**12**	3.790	3.957	1.044	*a*^0^*a*^0^*c*^−^	—	—
**30**	3.790	3.959	1.045	*a*^0^*a*^0^*c*^−^	1	1
**50**	3.790	3.958	1.044	*a*^−^*a*^−^*c*^−^	1.013	1.006
**80**	3.792 (L)	3.956 (L)	1.043 (L)	*a*^−^*a*^−^*c*^−^	1.221	1.040
3.796 (R)	3.947(R)	1.040 (R)				
**100**	3.792 (L)	3.954(L)	1.043 (L)	*a*^−^*a*^−^*c*^−^	1.275	1.102
3.807 (R)	3.924(R)	1.031 (R)				

*T*_c_ was normalized to that of the 30-nm film. For the lattice constants of the 80-nm and 100-nm films, the in-plane lattice constant was calculated based on volume-conservation. Refer to the text for more details.
