# Supplementary material for: Effects of strain relaxation in Pr0.67Sr0.33MnO3 films probed by polarization dependent X-ray absorption near edge structure
Source: Sci Rep. 2016 Jan 28;6:19886. doi: 10.1038/srep19886 (PMC4730223; doi:10.1038/srep19886)
Supplement: Supplementary Information [file srep19886-s1.pdf]

Supplementary Information

for

Effects of strain relaxation in $Pr_{0.67}Sr_{0.33}MnO_3$ films probed by polarization dependent X-ray absorption near edge structure

Bangmin Zhang,[1] Jingsheng Chen,[1] Ping Yang,[2] Xiao Chi,[2,3] Weinan Lin[1], T. Venkatesan,[1,3,4,5] Cheng-Jun Sun,[6,*] Steve M. Heald,[6] and Gan Moog Chow[1,*]

[1]*Department of Materials Science & Engineering, National University of Singapore, 117576, Singapore*

[2]*Singapore Synchrotron Light Source (SSLS), National University of Singapore, 5 Research Link, 117603 Singapore*

[3]*Department of Physics, National University of Singapore, 117542, Singapore*

[4]*NUSNNI-Nanocore, National University of Singapore, 117411, Singapore*

[5]*Department of Electrical & Computer Engineering, National University of Singapore, 117576, Singapore*

[6]*Advanced Photon Source, Argonne National Laboratory, Argonne, IL 60439, USA*

**Corresponding Authors' Email: cjsun@aps.anl.gov; msecgm@nus.edu.sgs*

S1: Mn *L* edge XLD

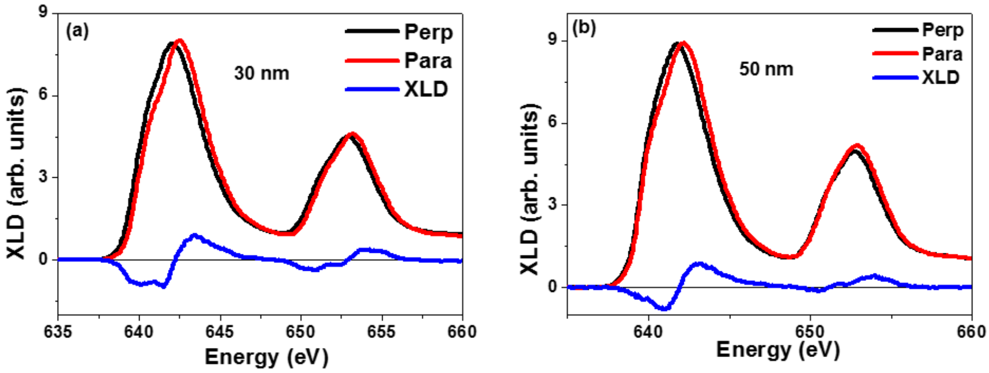

Figure S1: The Mn *L* edge XAS and correlated XLD for (a) 30-nm and (b) 50-nm PSMO films on (001) LAO substrate.

Mn ***L*** edge is a useful method to detect the strain effect on the electronic structure in manganite. In this work, Mn ***L*** edge XLD was collected in total electron yield (TEY) mode at room temperature for 30-nm and 50-nm PSMO films on (001) $LaAlO_3$ substrate. During the measurement, the electric field of linear polarized X-ray was either parallel to (red line) or perpendicular to (black line) the film plane and the difference between these two lines was recorded as XLD (blue line). This could give direct information on the orbital occupancy of Mn 3*d* orbitals. The absorption feature in the 639 eV is from the electronic excitation from the 2*p* orbital to the 3*d* orbital. As shown in the above figure, the $3d_{z^2}$ has a higher occupancy than that of $3d_{x^2-y^2}$ due to the elongation of out-of-plane lattice constant[1]. The strain-induced change on the crystal structure, including the lattice constant and octahedral rotation, directly affects the electronic structure and corresponding XLD absorption intensity. The change of the features in the 639 eV reflects the change of the strain-induced effects on the electronic structure. Compared to the ***K*** edge XANES in the main text, the ***L*** edge XANES was collected in TEY mode, yielding information from a few nanometers near the surface. The ***K*** edge XANES collected in the total fluorescence yield (TFY) mode is bulk sensitive, including contribution from the entire film. Hence, the

polarization dependent ***K*** edge XANES could provide complementary information to the ***L*** edge XLD.

S2: XRD curve fitting

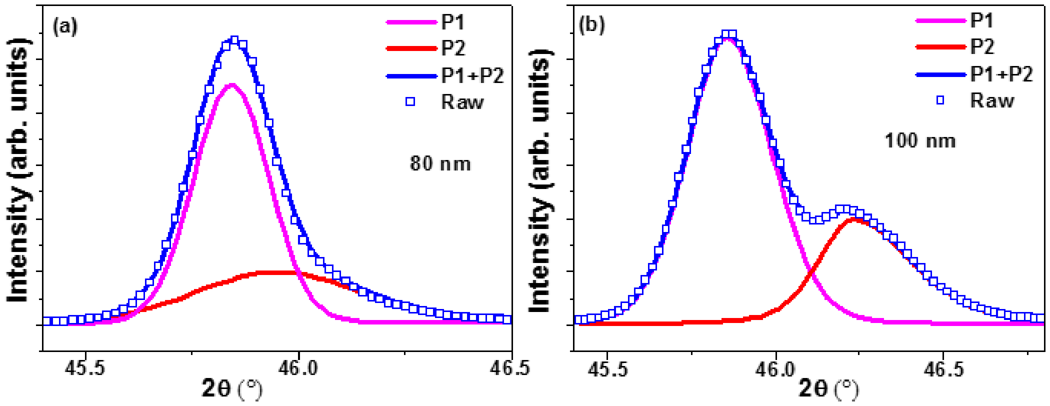

(c)

	80 nm		100 nm	
	P1	P2	P1	P2
c (Å)	3.956	3.947	3.955	3.924
Area ratio	0.685	0.315	0.694	0.306

Figure S2: Fitting of (002) peak of (a) 80-nm and (b) 100-nm PSMO films. The open square is the measured results, and the solid lines are fitting results. (c) Summary of fitting results.

The (002) peak of 80-nm and 100-nm films was fitted with two sub-peaks as shown above. The calculated out-of-plane lattice constant ***c*** and corresponding area ratio of each peak are summarized above. The in-plane lattice constant of each peak was calculated based on assumption of volume-conservation. In fitting the Curie temperature (discussed in the main text), the area ratio was used to calculate the averaged lattice constant of these two peaks.

S3: Resistivity Fitting

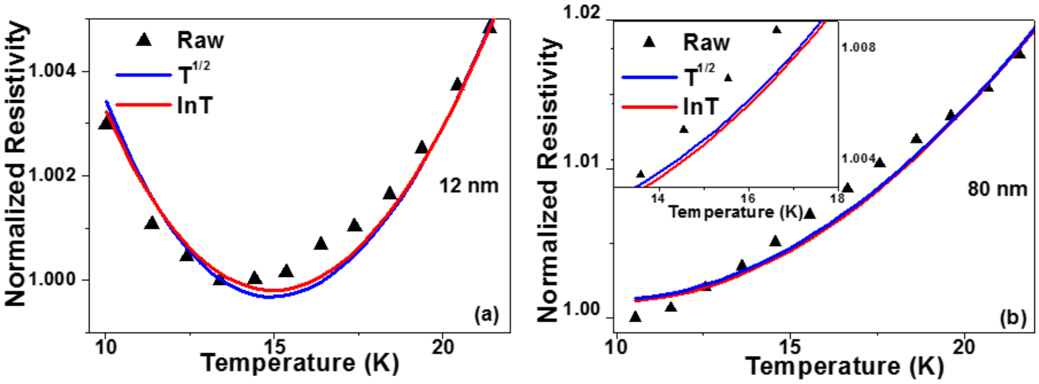

Figure S3: Fitting resistivity for (a) 12 nm and (b) 80 nm films at low temperature.

The above two figures shows the fitting details for the 12-nm and 80-nm PSMO films, respectively. The fitting was performed in the temperature range of 10 to 60 K. Only the part (10 to 22 K) with a large discrepancy between the fitting and measured results is shown above. For the 12-nm film, the logarithmic dependent equation (lnT, red line, $\chi^2 = 2.5\times10^{-7}$), fits the measured resistivity (black triangle) slightly better than the $T^{1/2}$ dependent equation ($T^{1/2}$, blue line, $\chi^2 = 3.1\times10^{-7}$). For the 80-nm film, the $T^{1/2}$ dependent equation ($T^{1/2}$, blue line, $\chi^2 = 7.8\times10^{-7}$), fits the measured resistivity (black triangle) slightly better than the logarithmic dependent equation (lnT, red line, $\chi^2 = 8.3\times10^{-7}$). The χ^2 values are plotted in Fig. 6d.

Similarly, for the 100-nm film $\chi^2(T^{1/2})$ is 4.62×10^{-8}, and $\chi^2(\ln T)$ is 4.74×10^{-8}. The absolute difference between the two χ^2 values is very small, probably indicating the decreasing role played by the QIE correction terms in both equations[2]. These two equations with the QIE correction terms may no longer be applicable in thick films.

References:

1. Pesquera, D. *et al.* Surface symmetry-breaking and strain effects on orbital occupancy in transition metal perovskite epitaxial films. *Nat. Commun.* **3**, 1189 (2012).
2. Maritato, L. *et al.* Low-temperature resistivity of $La_{0.7}Sr_{0.3}MnO_3$ ultra thin films: Role of quantum interference effects. *Phys. Rev. B* **73**, 094456 (2006).
